# Supplementary material for: Differential expression of MAGEA6 toggles autophagy to promote pancreatic cancer progression
Source: eLife. 2020 Apr 9;9:e48963. doi: 10.7554/eLife.48963 (PMC7164953; doi:10.7554/eLife.48963)
Supplement: Supplementary file 1. [file elife-48963-supp1.docx]

| **Key Resources Table** | | | | |
| --- | --- | --- | --- | --- |
| **Reagent type (species) or resource** | **Designation** | **Source or reference** | **Identifiers** | **Additional information** |
| gene (Homo sapiens) | MAGEA3 |  | GeneID: 4102 |  |
| gene (Homo sapiens) | MAGEA4 |  | GeneID: 4103 |  |
| gene (Homo sapiens) | MAGEA6 |  | GeneID: 4105 |  |
| gene (Homo sapiens) | MAGEA10 |  | GeneID: 4109 |  |
| gene (Homo sapiens) | MAGEA12 |  | GeneID: 4111 |  |
| strain, strain background (include species and sex here) | Athymic Nude-*Foxn1^nu^*  (Mus musculus; female) | Envigo | 069(nu)/070(nu/+)  RRID:  MGI:5652489 |  |
| strain, strain background (include species and sex here) | Stbl3 | ThermoFisher Scientific | C737303 |  |
| genetic reagent (Homo sapiens) | MAGEA3 | ThermoFisher  Scientific | IOH3602 | Ultimate™ ORF LITE Clones |
| genetic reagent (Homo sapiens) | MAGEA4 | ThermoFisher  Scientific | IOH11259 | Ultimate™ ORF LITE Clones |
| genetic reagent (Homo sapiens) | MAGEA6 | ThermoFisher  Scientific | IOH28024 | Ultimate™ ORF LITE Clones |
| genetic reagent (Homo sapiens) | MAGEA10 | ThermoFisher  Scientific | IOH5670 | Ultimate™ ORF LITE Clones |
| genetic reagent (Homo sapiens) | MAGEA12 | ThermoFisher  Scientific | IOH3031 | Ultimate™ ORF LITE Clones |
| cell line (Homo sapiens) | HPDE | PMID: 11073822 | RRID: CVCL_0P37 |  |
| cell line (Homo sapiens) | HPDE-iKRAS | PMID: 26806015 | RRID:  CVCL_YY28 | Established in the lab |
| cell line (Homo sapiens) | AsPC-1 | ATCC | CRL-1682;  RRID:  CVCL_0152 |  |
| cell line (Homo sapiens) | BxPC-3 | ATCC | CRL-1687;  RRID:  CVCL_0186 |  |
| cell line (Homo sapiens) | Capan-1 | ATCC | HTB79;  RRID:  CVCL_0237 |  |
| cell line (Homo sapiens) | Capan-2 | ATCC | HTB80;  RRID:  CVCL_0026 |  |
| cell line (Homo sapiens) | Panc-1 | ATCC | CRL-1469;  RRID:  CVCL_0480 |  |
| cell line (Homo sapiens) | MIA PaCa-2 | ATCC | CRL-1420;  RRID:  CVCL_0428 |  |
| transfected construct (Homo sapiens) | MAGEA6 shRNA #4 | Sigma-Aldrich | TRCN0000151826 | MISSION pLKO shRNA |
| transfected construct (Homo sapiens) | MAGEA6 shRNA # 10 | Sigma-Aldrich | TRCN0000155661 | MISSION pLKO shRNA |
| transfected construct (Homo sapiens) | ATG7 shRNA | Sigma-Aldrich | TRCN0000007584 | MISSION pLKO shRNA |
| transfected construct (Homo sapiens) | VPS34 shRNA | Sigma-Aldrich | TRCN0000196247 | MISSION pLKO shRNA |
| antibody | MAGEA3 (Rabbit monoclonal) | Abcam | ab223162  RRID:  AB_2827928 | 1:1000 |
| antibody | MAGEA4 (Rabbit polyclonal) | Abcam | ab76177;  RRID:  AB_1310427 | 1:1000 |
| antibody | MAGEA6 (Rabbit polyclonal) | Abcam | ab38495;  RRID:  AB_2138128 | 1:1000 |
| antibody | MAGEA10 (Rabbit polyclonal) | Abcam | ab83557;  RRID:  AB_1860232 | 1:1000 |
| antibody | MAGEA12 (Rabbit polyclonal) | Thermo Fisher Scientific | PA5-67682;  RRID:  AB_2691455 | 1:1000 |
| antibody | GAPDH (Rabbit polyclonal) | Santa Cruz Biotechnology | Sc-25778;  RRID:  AB_10167668 | 1:1000 |
| antibody | phospho-ERK1/2 (Rabbit polyclonal) | Cell Signaling Technology | 9101S;  RRID:  AB_331646 | 1:1000 |
| antibody | phospho-AMPK (Rabbit polyclonal) | Cell Signaling Technology | 2531S;  RRID:  AB_330330 | 1:1000 |
| antibody | phospho-p70 S6 kinase (Rabbit polyclonal) | Cell Signaling Technology | 9205S;  RRID:  AB_330944 | 1:1000 |
| antibody | phospho-AKT (Rabbit polyclonal) | Cell Signaling Technology | 9271S;  RRID:  AB_329825 | 1:1000 |
| antibody | SQSTM1/p62 (Mouse monoclonal) | Abcam | ab56416;  RRID:  AB_945626 | 1:1000 |
| antibody | Tp53 (Mouse monoclonal) | Cell Signaling Technology | 2524;  RRID:  AB_331743 | 1:1000 |
| antibody | V5 (Rabbit polyclonal) | Santa Cruz Biotechnology | sc-83849-R;  RRID:  AB_2019669 | 1:1000 |
| antibody | Ubiquitin (Rabbit polyclonal) | Abcam | Ab7780;  RRID:  AB_306069 | 1:1000 |
| antibody | LC3B  (Rabbit polyclonal) | Cell Signaling Technology | 2775S;  RRID:  AB_915950 | 1:1000 |
| antibody | LC3B  (Rabbit polyclonal) | Novus Biologicals | NB-100-2220;  RRID:  AB_10003146 | IF: 1:200  IHC: 1:4000 |
| recombinant DNA reagent | pLenti6.3  (plasmid) | Thermo Fisher Scientific | V53306 |  |
| sequence-based reagent | MAGEA6 RT#1 F | This paper | qRT-PCR primer | GGAGAAAATCTGGGAGGAGC |
| sequence-based reagent | MAGEA6 RT#1 R | This paper | qRT-PCR primer | TAGCTGGTTTCAATGAGGGC |
| sequence-based reagent | MAGEA6 RT#2 F | This paper | qRT-PCR primer | GAGGACTCCAGCAACCAAGA |
| sequence-based reagent | MAGEA6 RT#2 R | This paper | qRT-PCR primer | AGTACTGCCAATTTCCGACG |
| sequence-based reagent | MAGEA RT#3 F | This paper | qRT-PCR primer | CGTTGTGAGTTGGATAGTTGTGGAAA |
| sequence-based reagent | MAGEA RT#3 R | This paper | qRT-PCR primer | CTTCTGGGCATCCTTCAGCC |
| sequence-based reagent | L32 RT#4 F | This paper | qRT-PCR primer | CCTTGTGAAGCCCAAGATCG |
| sequence-based reagent | L32 RT#4 R | This paper | qRT-PCR primer | TGCCGGATGAACTTCTTGGT |
| sequence-based reagent | ATG7 RT#5 F | This paper | qRT-PCR primer | CAGTTTGCCCCTTTTAGTAGTGC |
| sequence-based reagent | ATG7 RT#5 R | This paper | qRT-PCR primer | CCAGCCGATACTCGTTCAGC |
| sequence-based reagent | VPS34 RT#6 F | This paper | qRT-PCR primer | CCTGGAAGACCCAATGTTGAAG |
| sequence-based reagent | VPS34 RT#6 R | This paper | qRT-PCR primer | CGGGACCATACACATCCCAT |
| commercial assay or kit | CellTiter-glo | Promega | G7572 |  |
| commercial assay or kit | SuperScript IV First-Strand Synthesis System | ThermoFisher Scientific | 18091050 |  |
| commercial assay or kit | Gateway™ LR Clonase™ II Enzyme mix | ThermoFisher Scientific | 11791020 |  |
| commercial assay or kit | KOD Hot Start DNA Polymerase | Millipore Sigma | 71086 |  |
| chemical compound, drug | BafA1 | Selleckchem | S1413 |  |
| chemical compound, drug | MG132 | Sigma-Aldrich | C2211 |  |
| software, algorithm | GraphPad Prism | GraphPad Prism | RRID: SCR_002798 |  |
| software, algorithm | FIJI (ImageJ) | FIJI (ImageJ) | RRID: SCR_002285 |  |
| other | DAPI | Thermo Fisher Scientific | D1306 |  |
|  |  |  |  |  |
