# Supplementary material for: Differential expression of MAGEA6 toggles autophagy to promote pancreatic cancer progression
Source: eLife. 2020 Apr 9;9:e48963. doi: 10.7554/eLife.48963 (PMC7164953; doi:10.7554/eLife.48963)
Supplement: Transparent reporting form [file elife-48963-transrepform.docx]

***eLife’s* transparent reporting form**

We encourage authors to provide detailed information *within their submission* to facilitate the interpretation and replication of experiments. Authors can upload supporting documentation to indicate the use of appropriate reporting guidelines for health-related research (see [EQUATOR Network](http://www.equator-network.org/%20)), life science research (see the [BioSharing Information Resource](https://biosharing.org/)), or the [ARRIVE guidelines](http://www.plosbiology.org/article/info:doi/10.1371/journal.pbio.1000412) for reporting work involving animal research. Where applicable, authors should refer to any relevant reporting standards documents in this form.

If you have any questions, please consult our Journal Policies and/or contact us: [editorial@elifesciences.org](mailto:editorial@elifesciences.org).

**Sample-size estimation**

- You should state whether an appropriate sample size was computed when the study was being designed
- You should state the statistical method of sample size computation and any required assumptions
- If no explicit power analysis was used, you should describe how you decided what sample (replicate) size (number) to use

Please outline where this information can be found within the submission (e.g., sections or figure legends), or explain why this information doesn’t apply to your submission:

For mice study, we planned the number of mice (biological replicates) per cohort based on the similar experiments previously performed in our laboratory and in literature. For each cohort, we injected the same number of mice with cells expressing target genes or GFP control. In a paradoxical approach, we injected same number of mice with cells expressing gene-targeting or non-targeting shRNAs. The number of samples analyzed can be found in the Figure Legends.

**Replicates**

- You should report how often each experiment was performed
- You should include a definition of biological versus technical replication
- The data obtained should be provided and sufficient information should be provided to indicate the number of independent biological and/or technical replicates
- If you encountered any outliers, you should describe how these were handled
- Criteria for exclusion/inclusion of data should be clearly stated
- High-throughput sequence data should be uploaded before submission, with a private link for reviewers provided (these are available from both GEO and ArrayExpress)

Please outline where this information can be found within the submission (e.g., sections or figure legends), or explain why this information doesn’t apply to your submission:

For mice study, we conducted each experiment once, including 3-5 mice with 2 injections per mice or 9 mice with 1 injection per mice, 6-10 tumors in total. Each injection is considered as a biological replicate.

We performed at least 2 independent experiments for each cell-based experiment and immunofluorescence staining experiment. qPCR analysis was performed in triplicates (technical replicates) and specific gene expression is presented as mean ± standard deviation in figures.

We did not remove any outliers and samples from analysis.

**Statistical reporting**

- Statistical analysis methods should be described and justified
- Raw data should be presented in figures whenever informative to do so (typically when N per group is less than 10)
- For each experiment, you should identify the statistical tests used, exact values of N, definitions of center, methods of multiple test correction, and dispersion and precision measures (e.g., mean, median, SD, SEM, confidence intervals; and, for the major substantive results, a measure of effect size (e.g., Pearson's r, Cohen's d)
- Report exact p-values wherever possible alongside the summary statistics and 95% confidence intervals. These should be reported for all key questions and not only when the p-value is less than 0.05.

Please outline where this information can be found within the submission (e.g., sections or figure legends), or explain why this information doesn’t apply to your submission:

Specific statistical analysis methods for the following experiments were indicated in:

Figure	1B – figure legend

2C – figure legend

3A – figure legend

5B – figure legend

6A, 6B, 6C, 6D – figure legend

6E – figure and figure legend

Figure 3-figure supplement 1

Figure 4-figure supplement 3

Figure 6-figure supplement 2

For mice experiment, raw data is presented in the figures, with each tumor represented by an individual dot.

No statistical analysis in all other experiments

(For large datasets, or papers with a very large number of statistical tests, you may upload a single table file with tests, Ns, etc., with reference to sections in the manuscript.)

**Group allocation**

- Indicate how samples were allocated into experimental groups (in the case of clinical studies, please specify allocation to treatment method); if randomization was used, please also state if restricted randomization was applied
- Indicate if masking was used during group allocation, data collection and/or data analysis

Please outline where this information can be found within the submission (e.g., sections or figure legends), or explain why this information doesn’t apply to your submission:

Samples were allocated into experimental group based on target gene expression and downregulation. Investigator was not blinded during group allocation, data collection and data analysis.

**Additional data files (“source data”)**

- We encourage you to upload relevant additional data files, such as numerical data that are represented as a graph in a figure, or as a summary table
- Where provided, these should be in the most useful format, and they can be uploaded as “Source data” files linked to a main figure or table
- Include model definition files including the full list of parameters used
- Include code used for data analysis (e.g., R, MatLab)
- Avoid stating that data files are “available upon request”

Please indicate the figures or tables for which source data files have been provided:

Summary table of all the clinical trial studies targeting MAGE-A antigens (to July 2018) is available in Supplementary File 2.

Summary table of all the mutations analyzed is available in Supplementary File 3.

Figure 1B-source data 1: Pool of genes with similar size range as MAGEA gene family members.

Figure 1B-source data 2: TCGA mutation frequency of 1000 genes randomly selected from Figure 1B-source data 1.

Figure 5B-source data 1: Source data of LC3B punta counting per cell in Figure 5B.
